# Mapping of control measures to prevent secondary transmission of STEC infections in Europe during 2016 and revision of the national guidelines in Norway

**DOI:** 10.1017/S0950268819001614

**Published:** 2019-09-09

**Authors:** L. Veneti, H. Lange, L. Brandal, K. Danis, L. Vold

**Affiliations:** 1 Division for Infection Control and Environmental Health, Norwegian Institute of Public Health, Oslo, Norway; 2 European Programme for Intervention Epidemiology Training (EPIET), European Centre for Disease Prevention and Control, (ECDC), Stockholm, Sweden; 3 Santé Public France, The French National Public Health Agency (SpFrance), Saint-Maurice, France

**Keywords:** Control measures, Europe, HUS associated, Shiga toxin-producing *E. coli*, STEC

## Abstract

In 2016, we reviewed preventive control measures for secondary transmission of Shiga-toxin producing *Escherichia coli* (STEC) in humans in European Union (EU)/European Free Trade Association (EEA) countries to inform the revision of the respective Norwegian guidelines which at that time did not accommodate for the varying pathogenic potential of STEC. We interviewed public health experts from EU/EEA institutes, using a semi-structured questionnaire. We revised the Norwegian guidelines using a risk-based approach informed by the new scientific evidence on risk factors for HUS and the survey results. All 13 (42%) participating countries tested STEC for *Shiga toxin* (*stx*) *1*, *stx2* and *eae* (encoding intimin). Five countries differentiated their control measures based on clinical and/or microbiological case characteristics, but only Denmark based their measures on routinely conducted *stx* subtyping. In all countries, but Norway, clearance was obtained with ⩽3 negative STEC specimens. After this review, Norway revised the STEC guidelines and recommended only follow-up of cases infected with high-virulent STEC (determined by microbiological and clinical information); clearance is obtained with three negative specimens. Implementation of the revised Norwegian guidelines will lead to a decrease of STEC cases needing follow-up and clearance, and will reduce the burden of unnecessary public health measures and the socioeconomic impact on cases. This review of guidelines could assist other countries in adapting their STEC control measures.

## Introduction

Shiga-toxin producing *Escherichia coli* (STEC), also called verocytotoxin-producing *E. coli* (VTEC), can lead to mild self-limiting diarrhoea, to haemorrhagic colitis, or the life-threatening haemolytic uremic syndrome (HUS). Children younger than 5 years old, immunocompromised persons and the elderly are most susceptible to STEC infections, and to severe complications, including HUS. Outbreaks of STEC infections in childcare facilities pose a particular public health threat [1]. Appropriate control measures to prevent secondary transmission of STEC infection in humans, apart from personal hygiene measures, include withdrawal from kindergarten and isolation within institutions. In addition, existence of public health guidelines and legislation to safeguard food and water against STEC contamination (production, preparation, storage) are also important, as is community education [2].

STEC can produce one or more of Shiga toxins (Stxs), of which two distinct types are known, *Stx1* and *Stx2*. Those are further divided into different subtypes. The majority of STEC also carry *eae*, a gene encoding the attaching and effacing (A/E) protein intimin. Both host-related factors (as for instance low age) and the presence of specific STEC virulence genes like *stx2*, particularly subtypes *stx2a* and *stx2d*, and *eae* have been associated with increased risk of HUS development [2–12].

Notification of STEC infections is mandatory in most countries in the European Union (EU) and the European Free Trade Association (EEA), except for four countries, where reporting is voluntary (Belgium, France, Italy, Luxembourg). In 2015, 28 European countries reported 6025 cases of STEC infection, including 5901 confirmed cases, resulting in a notification rate of 1.27 cases per 100 000 population. The case fatality was 0.2% among the 3352 confirmed cases for which this information was provided [13].

In Norway, STEC infection has been mandatory notifiable since 1995 to the Norwegian Surveillance System for Communicable Diseases (MSIS) at the Norwegian Institute of Public Health (NIPH). Detection, isolation and preliminary characterisation of STEC are done at medical microbiological laboratories throughout Norway. The majority of these laboratories distinguish *stx1* from *stx2*, but only a few subtype *stx* routinely. However, all medical microbiological laboratories are obligated to forward STEC isolates to the National Reference Laboratory (NRL) for enteropathogenic bacteria at NIPH for verification and further characterisation, including *stx* subtyping [14].

To prevent secondary transmission of STEC infection, NIPH has implemented strict control measures and follow-up for STEC cases belonging to risk groups for transmitting the disease. These risk groups include cases among children attending kindergarten, food handlers and staff in nursing homes and hospitals caring for immunocompromised persons. Cases belonging to these risk groups should remain home until they no longer shed STEC, ascertained by 3–5 consecutive negative stool specimens, taken at least 24 h apart (microbiological clearance).

Between 1996 and 2016, 1230 cases of STEC infection were notified in Norway, of which 31% (n = 382) were hospitalised and 7% (n = 84) developed HUS. In 2013–2016, the number of notified STEC cases in Norway increased (2013, 193 cases; 2014, 151 cases; 2015, 221 cases; 2016, 239 cases), largely attributed to the introduction of culture-independent diagnostic tests and unselective screening of STEC [15].

As an increasing number of STEC cases were identified, more STEC cases with less severe clinical symptoms were reported. The annual number of notified HUS cases remained low [15]. This growing number of less severe STEC cases challenged the existing system. The stringent precautions implemented to prevent transmission of STEC (3–5 negative stool samples) were onerous for the patients and their families, especially when clearance was prolonged. The impact was both (i) financial due to absence from work, either to avoid occupational risk for spreading the disease or to care for sick children with STEC infection, and (ii) psychological for families where children were kept away from kindergarten for an extended period of time.

Consequently, there was a need to revise the guidelines in Norway. Information regarding public health control measures for STEC infections, implemented by different European institutes, was not easily accessible and had never been compared across Europe. In autumn 2016, we conducted a survey to describe control measures implemented for STEC cases by different European public health institutes in order to inform the revision of control measures and follow-up of STEC cases in Norway. In this survey, we focused on reviewing control measures for the prevention of secondary human transmission including exclusion policy and follow-up of cases that required clearance.

## Methods

### Survey among EU experts

Survey participants were public health experts (key informants) responsible for the preparation of STEC prevention guidelines in European public health institutes in all 28 EU Member States and four EEA countries (Iceland, Liechtenstein, Norway and Switzerland). Respondents were invited through the ‘Food and Waterborne Diseases and Zoonoses Programme (FWD)’ organised by the European Centre for Disease Prevention and Control. Participation in this survey was voluntary.

### Definitions

We classified cases based on laboratory and clinical characteristics as follows: A: positive for *stx1* and *eae* with uncomplicated diarrhoea, B: positive for *stx1* and *eae* with bloody diarrhoea, C: positive for *stx2* and *eae* with uncomplicated diarrhoea, D: positive for *stx2* and *eae* with bloody diarrhoea, E: developed HUS with positive laboratory test for STEC, F: developed HUS with only clinical criteria.

### Data collection procedure

Two NIPH researchers conducted together telephone interviews using a semi-structured questionnaire on (i) national recommendations and harmonisation of the sub-national level guidelines for STEC, (ii) laboratory methods to test for STEC, (iii) control measures to prevent secondary transmission for cases and close contacts. For most institutes, we interviewed at least two key informants.

We piloted the survey in four institutes, after which, the FWD Coordination Committee revised the questionnaire.

### Data analysis

To describe the reported control measures for STEC, we performed qualitative analysis. We calculated proportions, using as the denominator the total number of participating countries or entities (for countries with different sub-national policies).

### Revision of the Norwegian guidelines for control measures of STEC infections

In autumn 2016, following the survey, we updated the STEC guidelines in Norway, taking into account the survey findings and the new evidence regarding the association between HUS and virulence profile of the STEC strains [2–12].

## Results

Overall, 14/32 (44%) countries responded to our request for participation. We could not include Germany as no national guidelines were available and each of the 16 German states had their own recommendations. The following 13 countries responded to our survey: Austria, Belgium (Brussels, Flanders and Wallonia), Denmark, Finland, France, Greece, Ireland, the Netherlands, Slovenia, Spain, Sweden, UK and Norway. Two responses were obtained from Belgium, reflecting sub-national policies; Brussels and Flanders (that followed the same guidelines) reported different guidelines from Wallonia. These were included as separate entities in the analysis (subsequently referred to as countries), bringing the total number of participants to 14 (participation rate 42%, 14/33).

### General information about national recommendations

Austria and Sweden based their responses on sub-national guidelines (that were considered representative) since national guidelines were not available. All other respondents reported that their national and sub-national guidelines were harmonised with no substantial differences. The Belgian regions of Brussels and Flanders had different policies than the Wallonia region. Spain reported that differences might have existed at the sub-national level guidelines which were more detailed than the national ones (Table 1).

**Table 1.** General information about the last update of STEC national recommendations and harmonisation with sub-national level guidelines, EU/EEA countries, survey 2016

Country	Last update	Reason of update	Differences in sub-national versus national level guidelines?
Austria	March 2016	Request from public health medical doctors for clearer recommendations	No national guidelines available (local guidelines from Styria)
Belgium, Brussels and Flanders	2015	Routine procedure	No
Belgium, Wallonia	2016	After outbreak in a kindergarten	No
Denmark	Sept. 2015	Laboratory methods have changed (virulence profiles associated with HUS)	No
Finland	2007	First version	No
France	2015	After HUS outbreak	No
Greece	2011	After E.coli outbreak in Germany	No
Ireland	2013	Routine procedure	No
The Netherlands	July 2016	Notification criteria changed	No
Slovenia	2016	Raise awareness among risk groups	No
Spain	2013	Routine procedure	Unknown (recommendations at sub-national level may be more detailed)
Sweden	2013	Routine procedure	No national guidelines available
UK	2016	Routine procedure	No
Norway	2013	Routine procedure	No

Eight (57%) respondents had updated their guidelines within the previous 2 years (2015–2016); the remaining six (43%) had updated them during 2007–2013. Ten (71%) respondents updated their guidelines following an outbreak (national or international) or as a routine procedure (Table 1). In 2015, Denmark updated their guidelines to take into account the new evidence of the association between HUS and different virulence profiles of STEC strains.

### Laboratory methods

The reference laboratory in all participating countries reported using PCR and serotyping for characterisation of STEC. Additionally, five used enzyme immunoassay and 10 performed whole genome sequencing (WGS). All examined STEC isolates by PCR for the presence of *stx1*, *stx2* and *eae*, as well as other virulence genes such as *ehxA*, *aggR* and *aaiC*. Additionally, 12 reference laboratories were able to subtype *stx1* and *stx2*, of which seven performed that routinely (Table 2).

**Table 2.** Laboratory methods for characterisation and verification of microbiological clearance of STEC available at the National Reference Laboratory in participant countries, EU/EEA survey 2016

Country	Laboratory method for characterisation of STEC strains									Laboratory methods for verification of microbiological clearance		
	PCR						Serotyping	Immunoassay *(stx*)	WGS	Isolation	PCR (*stx*)	Immunoassay
	*stx*	*stx1*	*stx2*	*stx1* subtyping	*stx2* subtyping							
Austria	R	R	R	NR	NR		R	NR	NR	NU	U	U
Belgium^a^	R	R	R	NR	NR		R	NA	NR	U	U	NU
Denmark	R	R	R	R	R		R	NA (but VCA)	R	U	U	NU
Finland	R	R	R	NR	NR		R	NA	R	U	U	NU
France	R	R	R	R	R		R	NA	NR	U	U	NU
Greece	R	R	R	R	R		R	R	NA	U	U	U
Ireland	R	R	R	NR	NR		R	NA	NA	U	U	NU
The Netherlands	R	R	R	NA	R only for *stx2f*	NA for ≠*stx2f*	R	R	U	U	NU	NU
Slovenia	R	R	R	NR	NR		R	NA	NA	U	U	NU
Spain	R	R	R	R	R		R	NR	NR	U	U	U
Sweden	R	R	R	R	R		R	NA	R	U	U	NU
UK	R	R	R	R	R		R	NA	R	U	U	NU
Norway	R	R	R	R	R		R	NR	NR	U	U	NU

R, available and routinely used; NR, available, but not routinely used; NA, not available; U, used; NU, not used; VCA, vero cell assay.

a This refers to the reference laboratory of Belgium. The same reference laboratory is operating for all three regions of Belgium (Wallonia, Brussels and Flanders)

All respondents were aware of the latest publications on the association between different STEC strains, virulence profiles and HUS [2–12]. However, Denmark was the only country using *stx* subtyping routinely to differentiate low-virulent STEC from high-virulent STEC. Denmark considered STEC with *stx2* subtypes *stx2a* and *stx2d* to be high-virulent, while STEC with *stx1*, *stx2b*, *stx2c*, *stx2e*, *stx2f*, *stx2g* were classified as low-virulent [16].

Different practices were reported from the participant countries regarding the laboratory methods used for microbiological clearance. Nine reference laboratories reported that they used isolation of STEC and PCR (*stx* genes) (Table 2).

### Risk groups and control measures recommended for STEC cases

In all participating institutes, implementation of control measures to prevent further primary and secondary cases always included hygiene and infection control recommendations to all cases and identification of the source or vehicle of the disease if possible. In addition, all cases and contacts were interrogated to determine whether they required exclusion from work/school and/or testing for microbiological clearance.

All institutes considered specific high-risk groups for secondary transmission of STEC infection (further details in Supplementary material, Part A). Distinct control measures were recommended for cases that belonged to high-risk groups, including exclusion and clearance criteria. All 14 (100%) respondents identified children aged <5 (or ⩽5) years old who attend kindergarten (pre-schools, nurseries or other similar child care or minding groups), food handlers and people who attend/work at day care or nursing homes as high-risk groups for secondary transmission of the disease. Other high-risk groups reported by the respondents were people unable to toilet themselves and maintain satisfactory hygiene (because of underlying complications) (50%), children aged <5 years old who do not attend kindergarten (43%), immunocompromised people (36%), children attending schools (with variation in ages) (29%), elderly who live in hospitals/nursing homes (21%), immunocompromised people who live in hospitals/nursing homes (21%) and elderly (⩾65 years old) (7%).

Recommendations for cases belonging to high-risk groups for secondary transmission included (i) exclusion from work/school/kindergarten, (ii) the number of consecutive negative stool samples needed for microbiological clearance, with variable intervals between sampling. All respondents mentioned that decisions about risk, exclusion and timing of microbiological clearance could depend on specific local circumstances and might differ in outbreak settings.

Nine (64%) respondents did not differentiate their control measures for cases belonging to categories of cases A–F (Group I countries) and the number of negative stool samples required for clearance ranged from 1 to 3 (Table 3). Five (36%) respondents differentiated their control measures for cases belonging to categories of cases A–F (Group II countries) and the number of negative stool samples ranged from 0 to 5 (Table 4). Only Denmark differentiated their control measures based on routinely *stx* subtyping of all STEC isolates. In all countries, but Norway, clearance was obtained with ⩽3 negative specimens.

**Table 3.** Microbiological clearance required for cases that belong to categories of STEC cases A–F^a^, countries that did not differentiate control measures (Group I), EU/EEA survey 2016

Country	No. of negative control samples	Hours interval of laboratory sampling	Start sampling^b^
Belgium, Brussels and Flanders	2	48	AS
Belgium, Wallonia	2	24	AS
Finland	3	24–48	AS and at least a week after 1st positive sample
France	2	48	AS
Greece	2	24	AS (If antibiotics, 48 h after the last dose of antibiotics)
The Netherlands	2	48	AS
Slovenia	2	24 (48 if prolonged diarrhoea)	AS (If prolonged diarrhoea, 48 h AS)
Spain	2	24	AS (If antibiotics, 48 h after the last dose of antibiotics)
Sweden	1	−	AS

a (A) Positive for stx1 and eae with uncomplicated diarrhoea, (B) positive for stx1 and eae with bloody diarrhoea, (C) positive for stx2 and eae with uncomplicated diarrhoea, (D) positive for stx2 and eae with bloody diarrhoea, (E) developed HUS with positive laboratory test for STEC, (F) developed HUS with only clinical criteria.

b AS: after symptoms of diarrhoea have ceased.

**Table 4.** Microbiological clearance required for cases that belong to categories of STEC cases A–F, countries that differentiated control measures (Group II), EU/EEA survey 2016

Category	Country		No. of negative control samples	Hours interval of laboratory sampling	Start sampling^a^
A. Positive for *stx1* and *eae* with uncomplicated diarrhoea	Austria		3	48	1 week AS (If antibiotics at least 3 days after last dose of antibiotics)
	Denmark		0	–	–
	UK		0	–	–
	Ireland		2	24	AS
	Norway	O103	5	24	2–3 days AS
		≠ O103	3	24	2–3 days AS
B. Positive for *stx1* and *eae* with bloody diarrhoea	Austria		3	48	1 week AS (If antibiotics at least 3 days after last dose of antibiotics)
	Denmark		0	–	–
	UK		2	24	AS
	Ireland		2	24	AS
	Norway	O103	5	24	2–3 days AS 2–3 days AS
		≠ O103	3	24	
C. Positive for *stx2* and *eae* with uncomplicated diarrhoea	Austria		3	48	1 week AS (If antibiotics at least 3 days after last dose of antibiotics)
	Denmark	*stx2a or stx2d*	2	Not specified	AS
		≠ *stx2a, stx2d*	0	–	–
	UK		2	24	AS
	Ireland		2	24	AS
	Norway		5	24	2–3 days AS
D. Positive for *stx2* and *eae* with bloody diarrhoea	Austria		3	48	1 week AS (If antibiotics at least 3 days after last dose of antibiotics).
	Denmark	*stx2a or stx2d*	2	Not specified	AS
		≠ *stx2a, stx2d*	0	–	–
	UK		2	24	AS
	Ireland		2	24	AS
	Norway		5	24	2–3 days AS
E. Develop HUS with positive laboratory test for STEC	Austria		3	48	1 week AS (If antibiotics at least 3 days after last dose of antibiotics).
	Denmark		2	Not specified	AS
	UK		2	24	AS
	Ireland		2	24	AS
	Norway		5	24	2–3 days AS
F. Develop HUS with only clinical criteria	Austria		1	–	1 week AS (If antibiotics at least 3 days after last dose of antibiotics)
	Denmark		2, if no other reason for HUS is found	Not specified	AS
	UK		2	24	AS
	Ireland		0	–	–
	Norway		5	24	2–3 days AS

a AS: after symptoms of diarrhoea have ceased.

### Exclusions and clearance for close contacts of cases that belong to high-risk groups

All countries recommended control measures for close contacts of cases that belonged to risk groups for secondary transmission of the disease. The participating countries defined a close contact as a person living in the same household as the index case or regularly shared food or toilet facilities with the index case during the infectious period. This could be extended to family members who frequently visited the household and childminders (further details in Supplementary material, Part B).

All countries, but Sweden, differentiated control measures for symptomatic and asymptomatic contacts. Sweden treated all contacts as cases and excluded them from work/school until they had microbial clearance.

Group I countries treated symptomatic close contacts belonging to high-risk groups for transmitting the disease as cases (Table 3). For countries in Group II, symptomatic contacts were excluded and clearance was confirmed after three negative stool samples (48 h interval) for Austria, three negative stool samples (24 h interval) for Norway, two negative stool samples (24 h interval) for Ireland and the UK and one negative stool sample for Denmark.

Regarding asymptomatic close contacts that belonged to high-risk groups for secondary transmission of disease, only three (21%) countries (Austria, Sweden and Norway) always required exclusion from work/school, screening and clearance. Further details about control measures that were implemented (exclusion, screening, clearance) for asymptomatic contacts are provided in Table 5.

**Table 5.** Screening/exclusion and microbiological clearance for asymptomatic close contacts of STEC cases belonging to high-risk groups, EU/EEA survey 2016

Country	Screening required always	Indication for screening/exclusion	Microbiological clearance	
			No. of negative control samples	Hours interval of sampling (start sampling^a^)
Austria	Yes	–	NR	NR
Belgium, Brussels and Flanders	No	Only if ⩾2 cases, screening of all contacts	NR	NR
Belgium, Wallonia	No	After ⩾1 case of O157 or ⩾2 cases of O26, O103, O11, O145, O121	NR	NR
Denmark	No	Test if children or food handler	1	– (AS)
Finland	No	Siblings in diapers whose pre-school age sibling is the index case	1	*–* (after 3 neg. samples of index case)
France	No	Siblings to the index case	NR	NR
Greece	No	⩾2 cases	NR	NR
Ireland	No	All household contacts that belong to high-risk group. All kindergarten contacts if transmission within kindergarten suspected (e.g. if further cases of HUS, bloody diarrhoea or a second STEC case identified)	2	24 (AS)
The Netherlands	No	Only children <5 years old	NR	NR
Slovenia	No	In case of outbreak, in case of contacts who work with food or in health care	NR	NR
Spain	No	Depends on the laboratory (some request samples)	NR	NR
Sweden	Yes	–	1	– (AS)
UK	No	Depends on local authorities (some request samples). But all class/nursing home is tested If there is a symptomatic contact to kindergarten/nursing homes	2	24 (AS)
Norway	Yes	–	3	24 (AS and AS of index case)

NR, not reported; −, not applicable.

a AS: after symptoms of diarrhoea have ceased.

### Revision of the Norwegian guidelines for control measures of STEC infections

Until autumn 2016, the Norwegian recommendations were strict compared with other European countries. In particular, for some cases (Table 4, e.g. cases with *stx2*), Norway requested five negative stool samples for microbiological clearance, almost double compared with other countries. In the revised autumn 2016 recommendations, Norway distinguishes control measures for high-virulent STEC and low-virulent STEC to better target infection control and follow-up of cases. The differentiation is primarily based on the *stx* profile and clinical outcome. Only cases with high-virulent STEC infections who belong to high-risk groups for secondary transmission of disease are followed-up.

Based on the revised guidelines, cases with high-virulent STEC infection are determined by microbiological and clinical information and are those: (i) positive for *stx2* subtypes *2a*, *2c*, *2d*, or (ii) positive for *stx1* subtype *1a* in a patient ⩽5 years with bloody diarrhoea, or (iii) notified as a HUS-patient, or (iv) negative for *stx*, but *eae*-positive *E. coli* strain (STEC-LST) with a genotype previously seen in a HUS case.

Cases with low-virulent STEC infection are those positive for *stx1* (except ⩽5 years old with bloody diarrhoea and *stx1a*) or *stx2* subtypes *2b*, *2e*, *2f* and *2g*.

**Fig. 1.**
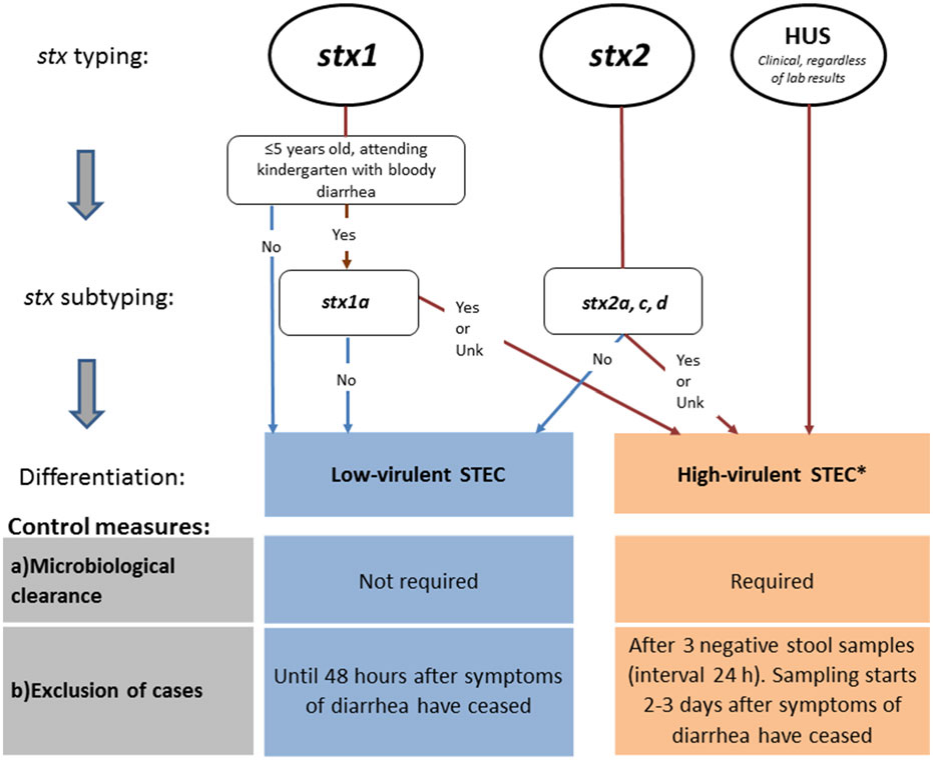
Revised Norwegian STEC guidelines, autumn 2016. Differentiation of control measures based on *stx* profile. ^a^STEC cases negative for stx, but eae-positive *E. coli* strain (STEC-LST) with a genotype (MLVA-type) previously seen in a HUS case in Norway are also classified as high-virulent STEC.

Cases with high-virulent STEC infection are excluded until microbiological clearance, confirmed by three consecutive negative stool specimens taken 24 h apart, with the first specimen taken 2–3 days after recovery. Cases with low-virulent STEC infection can return to work/kindergarten 48 h after recovery from diarrhoea without microbiological testing (Fig. 1).

Following the revised Norwegian guidelines, control measures to prevent secondary transmission are initiated upon detection of a case that could be classified as having a high-virulent STEC infection determined by clinical information and preliminary microbiological results from the medical microbiological laboratories (*stx1* in children ⩽5 years with bloody diarrhoea or detection of *stx2*). In Norway, the majority of the medical microbiological laboratories can distinguish between *stx1* and *stx2*, but do not perform *stx* subtyping. The prevention measures are maintained if further characterisation and *stx* subtyping of the STEC strain at the NRL confirm that the case carries a high-virulent STEC. If a low-virulent STEC is defined, the already initiated control measures are downgraded (Fig. 1). In addition, control measures for cases with no *stx* profile available are maintained and follow the guidelines of high-virulent infections.

Close contacts of cases with high-virulent STEC infection that belong to risk groups for secondary transmission of the disease are excluded from work/kindergarten regardless of their own symptoms. Exclusion lasts for the duration of the diarrhoea in the index patient, and until the close contact has provided one negative faecal sample. No exclusion or follow-up of close contacts of cases with low-virulent STEC infection is recommended.

### Implications of the new guidelines

Applying the new guidelines to the 212 STEC cases [*Note*: *During 2007–2016, 42% of the high-virulent STEC and 0.9% of the low virulent STEC were (non-sorbitol fermenting) STEC 0157. In 2016, 27% of the high-virulent STEC and 0% of the low virulent STEC were (non-sorbitol fermenting) STEC 0157*.], tested during 2016 where the *stx* profile was available (100 *stx1*, 112 *stx2*), 44% (94/212) of the cases would have been classified as infected with a low-virulent STEC based only on the *stx1* (taking in account age and clinical information) and *stx2* distinction. Of the 100 *stx1* cases, 64 were above 5 years old and 30 cases were under 5 years old but did not have bloody diarrhoea (number of children under 5 years with bloody diarrhoea = 6).

For the remaining 118 cases (112 *stx2* and 6 *stx1* in children under 5 years old with bloody diarrhoea), further *stx* subtyping was required to categorise high- *vs.* low-virulence but only 73 (62%) had information available regarding *stx* subtype (69 among stx2 and four stx1). Of these with known *stx* subtype, 53% (39/73) would have been classified as carrying a low-virulent STEC based on the *stx* subtype (39 among *stx2* and none *stx1*). This suggests that 63% of all cases (133/212) tested in 2016 would not have required follow-up under the revised guidelines. The proportion of the cases that would not have required follow-up under the revised guidelines becomes 80% when taking into account only the 167 cases with sufficient virulence data (133/167). In contrast, following the previous guidelines, only 19% (41/212) of cases with a STEC infection did not require follow-up.

From the sampling date of the fecal sample until the isolate is received at NRL, it takes approximately 9 days. In between, the medical microbiological laboratories have identified and characterised the STEC (*stx1* and *stx2*). At NRL, the *stx* subtyping is performed within 2–3 days.

## Discussion

The survey indicated that national STEC guidelines existed in 77% of the participating EU/EEA countries, with sub-national guidelines being harmonised. Discrepancies among recommendations of different countries existed for exclusions and microbiological clearance of cases. Denmark was the only country who distinguished their control measures based on routinely conducted *stx* subtyping. Incidence of STEC infections varied among the participant countries, but no relation between the incidence and the level of strictness of the national recommendations has been identified [13]. The follow-up of cases and both asymptomatic and symptomatic close contacts, regarding exclusion periods and microbiological clearance, also varied among countries.

The different practices seemed to be based on country-specific experience, as precise scientific evidence regarding the duration of shedding is lacking. Previously, periods of shedding STEC were reported to range between 5 and 98 days [17–22]. The observed heterogeneity among different countries may reflect uncertainty regarding the effectiveness of public health measures, but also pragmatic (e.g. laboratory capacity, logistic constrains), economic or legal constraints. This variation in country policies highlights the need for more scientific evidence and research regarding microbiological clearance, including laboratory methods used (PCR more sensitive than isolation), duration of STEC shedding and differences in shedding based on STEC serotype and *stx*-subtype.

This survey, the new scientific evidence on risk factors for HUS [2–12] and the experience from Denmark [16] assisted Norway in the revision of the national guidelines. The newly revised guidelines differentiate control measures and follow-up between cases with high-virulent and low-virulent STEC infection. Furthermore, the number of stool samples for microbiological clearance has been reduced (from five to three for cases with high-virulent STEC and from three to zero for cases with low-virulent STEC). As of February 2019, no outbreaks or secondary cases have been reported linked to an index case with low-virulent STEC infection that returned to kindergarten/work without microbiological clearance, after implementation of the new guidelines.

Following the newly revised guidelines, only 20% of the reported STEC cases with sufficient virulence data available would have required follow-up in 2016. Therefore, the implementation of the revised guidelines is expected to lead to a decrease in the number of STEC cases needing clearance, reducing the socioeconomic impact on cases and their families. This will also allow better use of time and resources on STEC surveillance and prevention both by NIPH and the municipal medical officers.

The above seems especially relevant, as an increase in the number of reported STEC cases is expected in Europe in the near future, mainly due to changes in diagnostic methods and screening procedures. Culture-independent diagnostic tests, like PCR, have increased sensitivity compared to traditionally culturing methods. Additionally, screening all faecal samples for STEC using multiplex PCR panels detecting a number of enteropathogenic bacteria, instead of a selective diagnostic approach based on clinical or epidemiological criteria, will increase the incidence of STEC cases, especially for cases with low-virulent STEC [15]. This increase will reinforce the need for revision of country-specific guidelines to allow better use of resources on control measures regarding STEC infections. This overview of STEC control measures around Europe may assist other countries in making decisions for improvements of their control measures and follow-up of STEC cases.

Some discrepancies were observed between Denmark and the new guidelines in Norway regarding the distinction between cases with high-virulent and low-virulent STEC infections. First, Norway defines children ⩽5 years old who attend kindergarten, have bloody diarrhoea and carry *stx1* subtype *1a* as a case with high-virulent STEC. These cases are excluded from kindergarten until microbiological clearance, to ensure that children with severe symptoms (bloody diarrhoea) are isolated in order to prevent the potential spread of severe disease in kindergartens. Second, STEC carrying *stx2c* is included among the cases with high-virulent STEC infection in Norway. The *stx2c* gene is mainly detected in non-sorbitol fermenting O157:H7 STEC, a bacterium seen in patients with higher rate of hospitalisation compared to their non-O157 counterparts. However, *stx2c* was not previously associated with HUS [6]. It is important to emphasise that the Norwegian categorisation scheme for differentiating high- and low-virulent STEC will require periodically evidence-based revision in light of new epidemiological and microbiological information.

### Limitations

Our survey suffers from some limitations. The low participation (42%) in our survey may not permit a complete overview of the public health measures implemented for STEC infection throughout Europe. However, the survey included countries from various geographical areas in Europe and with different experiences with control measures of cases with a STEC infection. In addition, economic considerations may also play a role in the recommendations proposed in each country, but this was not addressed in our study.

### Conclusions and recommendations

The survey and the new scientific evidence on risk factors for HUS informed the revision of the Norwegian guidelines, with the virulence profile of the STEC strains as well as clinical outcome informing the need for relevant control measures to prevent secondary human transmission. The implementation of those guidelines leads to a decrease in the number of STEC cases needing follow-up and clearance, reducing the socioeconomic impact on cases and their families, as well as the burden for public health professionals. This survey and the guideline revision could assist other countries to adapt their control measures for STEC infections. We recommend continuing follow-up only of cases infected by high-virulent STEC using a risk-based approach to reduce the burden of unnecessary public health measures. Further research regarding microbiological clearance, duration of STEC shedding and differences in shedding based on STEC serotype and *stx-*subtype would also be needed to increase available evidence that will guide better implementation of control measures.

## Supplementary material

For supplementary material accompanying this paper visit https://doi.org/10.1017/S0950268819001614. click here to view supplementary material
