# Supplementary material for: Mapping of control measures to prevent secondary transmission of STEC infections in Europe during 2016 and revision of the national guidelines in Norway
Source: Epidemiol Infect. 2019 Sep 9;147:e267. doi: 10.1017/S0950268819001614 (PMC6805742; doi:10.1017/S0950268819001614)
Supplement: Supplementary file 1 [file S0950268819001614sup001.docx]

*Epidemiology and Infection*

*Mapping of control measures to prevent secondary transmission of STEC infections in Europe during 2016 and revision of the national guidelines in Norway.*

*L. Veneti, H. Lange, L. Brandal, K. Danis, L. Vold*

***Supplementary material***

**Part A: Risk groups for ongoing transmission**

All 14 respondents identified children aged <5 (or ≤ 5) years old who attend kindergarten (pre-schools, nurseries or other similar child care or minding groups), food handlers, and people who attend/work at day cares or nursing homes as high risk groups for transmitting the disease. Further details are provided at the supplementary Table S1.

Supplementary Table S1: Risk groups for ongoing transmission.

| **Country** | **Risk groups** | | | | | | | | | | |
| --- | --- | --- | --- | --- | --- | --- | --- | --- | --- | --- | --- |
|  | Children < 5 years old who attend kindergarten | Children < 5 years old who do not attend kindergarten | Children attending school (with variation in ages) | Food handlers | Elderly | Elderly who live in hospitals/ nursing homes | Immunocompromised persons | Immunocompromised persons who live in hospitals/ nursing homes | People who work/attend day-cares or nursing homes | People unable to toilet themselves | Other, specify |
| Austria | X |  | X | X |  | X |  |  | X |  | People who work/live in dormitories |
| Belgium^(a)^ | X |  |  | X |  |  | X |  | X | X |  |
| Denmark | X | X |  | X |  |  |  | X | X |  |  |
| Finland | X |  | X | X |  |  |  |  | X |  | People who work with newborns, under school aged, or work with water plant |
| France | X | X | X | X | X | X | X | X | X | X |  |
| Greece | X | X |  | X |  |  | X |  | X |  |  |
| Ireland | X |  |  | X |  |  |  |  | X | X | Health care workers |
| Netherlands | X | X |  | X |  |  | X |  | X | X |  |
| Slovenia | X | X | X | X |  | X |  | X | X |  | Elderly who live with children that attend school, immunocompromised who live with children that attend school or live with other immunocompromised people |
| Spain | X |  |  | X |  |  |  |  | X |  | People with poor personal hygiene or people without proper facilities for hand washing at work, school or at home |
| Sweden | X^(b)^ |  |  | X |  |  |  |  | X |  |  |
| United Kingdom | X | X |  | X |  |  |  |  | X | X |  |
| Norway | X^(c)^ |  |  | X |  |  |  |  | X | X |  |

(a): The risk groups are the same for all three regions of Belgium (Wallonia, Brussels and Flanders)

(b): In Sweden, children <6 years old who attend school

(c): In Norway, children ≤ 5 years old

**Part B: Close contacts of cases that belong to high risk groups**

All countries recommended control measures for close contacts (of cases) that belonged to risk groups for transmitting the disease. In this study a close contact was defined as a person living in the same household as the index case or regularly shared food or toilet facilities with the index case during the infectious period. This could be extended to family members who frequently visited the household and childminders. Further details are provided at the supplementary Table S2.

Supplementary Table S2: Definition of close contacts.

| **Country** | **Definition of close contacts** |
| --- | --- |
| Austria | Household (parents, siblings) |
| Belgium* | Family members, classmates, teachers, nurses taking care of patients |
|  |  |
| Denmark | Family, household members, children at the same class in kindergarten |
|  |  |
| Finland | Household members, school or kindergarten friends, same institution, grandparents taking care or grandchildren |
|  |  |
| France | Person with a high risk of exposure to faeces of a person infected by STEC (family members living in the same house, childminder or equivalent) |
|  |  |
| Greece | Family members, partners, close friends (classmates, teachers in kindergarten if there are ≥ 2 cases) |
|  |  |
| Ireland | Household contacts and kindergarten contacts |
|  |  |
| Netherlands | Family members, not clear in recommendations |
|  |  |
| Slovenia | Family members, sex partners, children and teachers at same class in kindergarten |
|  |  |
| Spain | Any close contact, not specified |
| Sweden | Family and household |
| United Kingdom | Family members and close friends (for children who play together) |
| Norway | Household members, people who spent time with cases (grandparents, partners, babysitters) |
|  |  |

**Note: The same definition of close contacts is used from all three regions of Belgium (Wallonia, Brussels and Flanders)*
